# Clinical features of patients with stress-induced cardiomyopathy associated with renal dysfunction: 7 case series in single center

**DOI:** 10.1186/1471-2369-14-213

**Published:** 2013-10-07

**Authors:** Min Ji Shin, Harin Rhee, Il Young Kim, Byeong Yun Yang, Sang Heon Song, Dong Won Lee, Soo Bong Lee, Ihm Soo Kwak, Jung Hyun Choi, Eun Young Seong

**Affiliations:** 1 Department of Internal Medicine, Division of Nephrology, Pusan National University School of Medicine, Gudeok-ro 179 Seo-gu, Busan, Republic of Korea; 2 Department of Internal Medicine, Division of Cardiology, Pusan National University School of Medicine, Gudeok-ro 179 Seo-gu, Busan, Republic of Korea

**Keywords:** Stress cardiomyopathy, Acute kidney injury, Hemodialysis

## Abstract

**Background:**

Stress-induced cardiomyopathy (sCMP) is characterized by transient wall-motion abnormalities involving the left ventricular apex and mid-ventricle that are precipitated by emotional or physical stress. As the heart and kidney influence each other’s function through bidirectional pathways, sCMP can induce renal dysfunction or be induced by renal dysfunction. This study reviewed the clinical characteristics and outcomes of patients with confirmed sCMP associated with renal dysfunction.

**Methods:**

We conducted a retrospective analysis of the medical records of all patients from our institution who were diagnosed with sCMP from March 2010 to April 2012. Each patient’s demographic characteristics, presenting symptoms, triggering events, electrocardiographic characteristics, laboratory data, echocardiographic study findings, cardiac catheterization data, and outcomes were reviewed.

**Results:**

Among 30 patients who were diagnosed with sCMP, 7 patients had associated renal dysfunction. Three patients were on maintenance hemodialysis (HD) and 4 patients had acute kidney injury (AKI). Their mean ejection fraction was 35.2% at initial echocardiography, and 57.2% at follow-up echocardiography. Pericardial effusion was detected in all HD patients initially; these patients were treated with intensive HD for suspected under-dialysis status. In patients with AKI, the mean peak serum creatinine was 4.17 mg/dL. Two patients were treated with continuous renal replacement therapy. One patient required maintenance HD, and 1 patient died. Two patients had full renal recovery to their baseline renal function at 7 and 14 days.

**Conclusions:**

Patients with renal dysfunction including those with AKI and those undergoing HD can develop sCMP, renal function must be closely monitored in patients with sCMP. Additionally, it should be considered that patients on HD who develop sCMP may be under-dialyzed.

## Background

Stress-induced cardiomyopathy (sCMP)-also known as takotsubo cardiomyopathy, broken heart syndrome, or transient left apical ballooning syndrome-usually presents as transient left ventricular dysfunction. This syndrome mimics an acute coronary syndrome, electrocardiograms (ECG) often reveal ST elevations, T wave inversion, and Q waves. Cardiac enzymes are also elevated but the coronary arteries are essentially normal. To date, there have been more than 1000 reports published on sCMP. It is estimated that sCMP accounts for 2% of all suspected acute coronary syndromes with 90% of the cases being in postmenopausal women. In all, 39%-80% of patients provide a history of a recent severe emotional or physical stressor [1,2]. Classic takotsubo cardiomyopathy is characterized by transient hypokinesia of the apical portion of the left ventricle with compensatory hyperkinesias of the basal walls, resulting in apical ballooning. Recently, reverse or inverted takotsubo cardiomyopathy has also been identified with the hyperdynamic apex and akinesia of the base of left ventricular wall [3,4]. It is generally resolved within days to weeks after initial presentation, and the overall prognosis seems to be favorable, although isolated cases of death have been reported.

Cardiorenal syndrome (CRS) is a disorder of the heart and kidney, whereby acute or long-term dysfunction in one of these organs may induce acute or long-term dysfunction of the other. Changes in the renin-angiotensin-aldosterone system, the sympathetic nervous system, an imbalance between nitric oxide and reactive oxygen species, and inflammation are the cardiorenal connectors in the development of CRS [5]. Similar to that observed in CRS, sCMP can induce renal dysfunction or be induced by renal dysfunction. However, there has been only 1 case report of sCMP with acute kidney injury (AKI) [6]. In this report, we reviewed a case series of 7 patients with confirmed sCMP associated with renal dysfunction including prevalent hemodialysis (HD) or acute kidney injury.

## Methods

We conducted a retrospective analysis of the medical records of all patients from our institution who were diagnosed with sCMP from March 2010 to April 2012. Patients who met the following criteria of sCMP were included: (1) development of typical takotsubo or inverted takotsubo on echocardiography after emotional or physical stress; (2) no evidence of obstructive epicardial coronary artery disease on coronary angiography (CAG); for those patients who could not undergo CAG, follow-up echocardiography showing improvement of left ventricular function was used to demonstrate the absence of coronary artery obstruction; (3) new ECG abnormalities, including ST-segment elevation, T-wave inversion, or arrhythmia and elevation in troponin I. We identified 30 patients with sCMP. Of these, 7 patients had associated renal dysfunction. Three patients were on maintenance HD with anuric state and 4 patients had AKI. AKI was diagnosed according to the RIFLE criteria, which can be used to classify patients with renal dysfunction according to the degree of impairment into “Risk,” “Injury,” “Failure,” “Sustained loss,” and “End-stage renal disease” categories [7]. Each patient’s demographic characteristics, presenting symptoms, triggering events, ECG characteristics, laboratory data, echocardiographic study findings, cardiac catheterization data, and outcomes were reviewed. Three patients (42.8%) were women and the mean age was 65 years (range, 53 to 77 years). The protocol was approved by the institutional review board of Pusan National University Hospital (E-2012059). Informed consent was exempted by the board because of the retrospective nature of the study.

## Results

**Figure 1:**
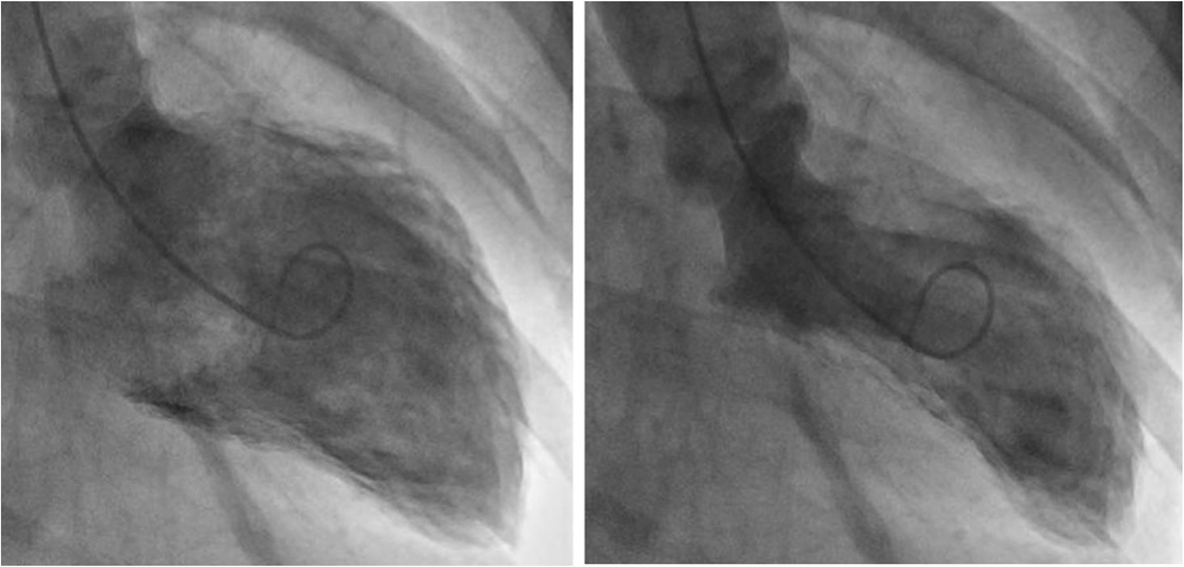
Left ventriculogram in diastole (Left) and systole (Right) of stress-induced cardiomyopathy showing apical ballooning of the left ventricle.

**Figure 2:**
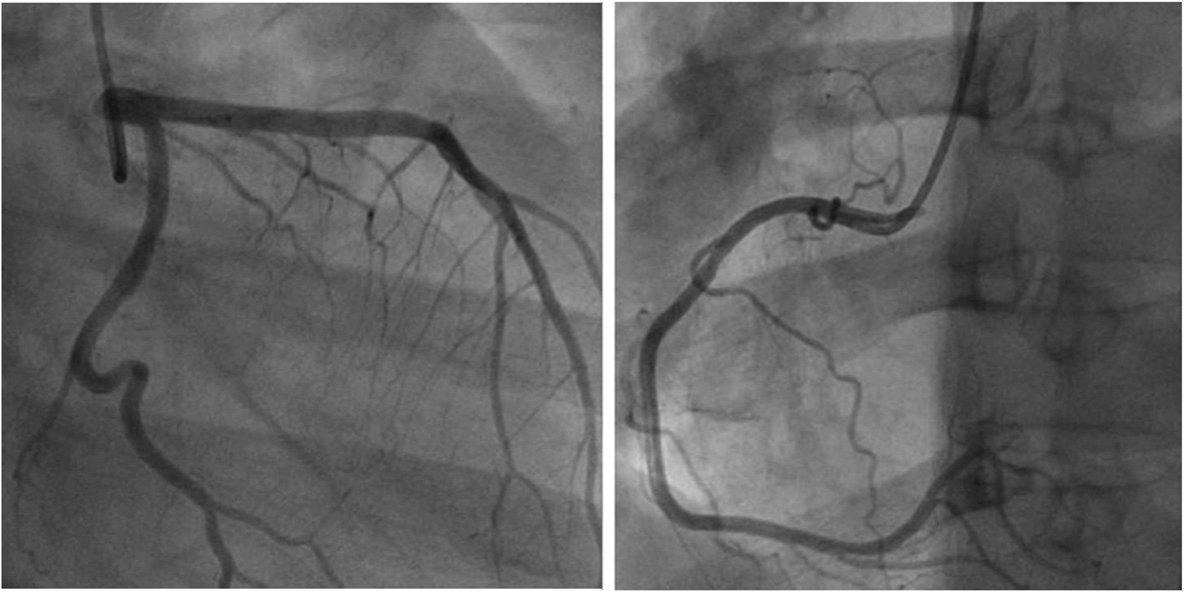
Angiogram showing normal coronary arteries.

Clinical characteristics and laboratory findings of the 4 patients with AKI and sCMP are listed in Tables 1 and 2. Three patients were men and the age range was 53 to 77 years. Two patients had diabetes mellitus and atrial fibrillation, and 1 patient had anti-neutrophil cytoplasmic autoantibody (ANCA)-associated rapidly progressive glomerulonephritis (RPGN). The fourth was a patient with pyriform sinus cancer who underwent adjuvant chemotherapy. On admission, only 1 patient presented with chest pain; the remaining 3 patients presented with dyspnea. The triggering event in all 4 patients was physical stress, which consisted of pneumonia and hypoxemia in 2 patients, infectious colitis in 1 patient, and RPGN in 1 patient. The ECG findings on admission were diffuse T-wave inversion in leads V1 to V6 in 2 patients and ST-segment elevation in the precordial leads in 1 patient. The second patient had only QTc prolongation (QTc = 501 ms). All patients indicated a small increase in cardiac enzyme levels. On Echocardiography at presentation, 3 patients showed the typical takotsubo pattern with left ventricular dysfunction (Figure 1) and the other patient had an inverted takotsubo pattern with left ventricular dysfunction. The mean ejection fraction (EF) was 36.2% (range, 30-48%). Three patients were evaluated with follow-up echocardiography and showed improvement of left ventricular function within a few weeks (range, 2-4 weeks). On follow-up echocardiography of 3 patients, the mean EF was 59.0% (range, 52-68%), and no regional wall motion abnormalities were noted. The second patient underwent CAG and indicated no evidence of obstructive epicardial coronary artery disease (Figure 2).

**Table 1:** Clinical characteristics of patients with acute kidney injury and stress-induced cardiomyopathy

**Patient No.**	**Underlying disease**	**Presenting symptom**	**Triggering event**	**RIFLE classification**	**Renal replacement therapy**	**Outcomes**
1	DM, HT, AF	Chest pain	Infectious colitis	Failure	CRRT	Recovery to baseline renal function and no recur of sCMP
2	DM, AF	Dyspnea	Pneumonia	—	CRRT	In-hospital death from recurrent VT
3	ANCA-RPGN	Dyspnea	ANCA-RPGN	ESRD	Conventional HD	Maintenance HD and no recur of sCMP
4	Pyriform sinus cancer	Dyspnea	Peumonia	Injury	No	Recovery to baseline renal function and no recur of sCMP

*DM* diabetes mellitus, *HT* hypertension, *AF* atrial fibrillation, *ANCA-RPGN* anti-neutrophil cytoplasmic autoantibody-associated rapidly progressive glomerulonephritis, *ESRD* end-stage renal disease, *CRRT* continuous renal replacement therapy, *HD* hemodialysis, *sCMP* stress-induced cardiomyopathy, *VT* ventricular tachycardia.

**Table 2:** ECG characteristics, laboratory data, echocardiographic studies, cardiac catheterization data of patients with acute kidney injury and stress-induced cardiomyopathy

**Patient No.**	**ECG abnormality**	**Echocardiographic abnormality at presentation**	**Peak troponin I (ng/mL)**	**Coronary angiography**	**EF at follow-up**	**hs-CRP (mg/dL)**	**Baseline SCr (mg/dL)**	**Peak SCr (mg/dL)**	**Follow-up SCr (mg/dL)**	**Time to recovery of SCr (day)**
1	T-wave inversion	Typical takotsubo, EF = 33%	0.15	NA	57 %	12.26	1.0	3.76	1.03	7
2	QTc prolongation	Inverted takotsubo, EF = 30%	0.38	Normal	NA	9.61	0.73	3.45	—	—
3	T-wave inversion	Typical takotsubo, EF = 34%	3.74	NA	52%	2.13	1.44	7.29	—	—
4	ST-segment elevation	Typical takotsubo, EF = 48%	3.56	NA	68%	19.1	0.9	2.19	1.02	14

*ECG* electrocardiography, *EF* ejection fraction, *NA* not available, *hs-CRP* high sensitive C-reactive protein, *SCr* serum creatinine.

In all 4 patients, the mean baseline serum creatinine was 1.02 mg/dL and the mean serum creatinine at the time of initial echocardiography was 3.0 mg/dL. The first patient’s peak serum creatinine level was 3.76 mg/dL. As this patient had unstable blood pressure with inotropic agent use and decreased urine volume (<400 mL/day), continuous renal replacement therapy (CRRT) was initiated, using the mode of continuous venovenous hemodiafiltration. She was treated with CRRT for 96 hours. After the CRRT was discontinued, she entered the polyuric stage and her renal function returned to baseline within approximately 7 days after diagnosed AKI. She exhibited normal renal function and no recurrence of sCMP at the last examination performed in the nephrology department. Therefore, the degree of renal impairment was “Failure,” according to the RIFLE criteria. The second patient had a maximum creatinine level of 3.45 mg/dL with decreased urine volume (urine volume <300 mL/day) and unstable blood pressure with inotropic agent use; therefore, CRRT was initiated. However, he died during the hospital period. His death was attributed to recurrent ventricular tachycardia. The third patient had biopsy-proven ANCA-associated RPGN and received steroid pulse therapy and cyclophosphamide therapy along with conventional hemodialysis. Despite the immunosuppressive regimen, his renal function did not improve, and therefore, maintenance hemodialysis was initiated. He did not exhibit any recurrence of sCMP at his last follow-up examination. The degree of renal impairment was “End-stage renal disease” according to the RIFLE criteria. In the fourth patient, the serum creatinine level reached to 2.19 mg/dL and then decreased to baseline levels after 14 days, with no recurrence of sCMP. The degree of renal impairment according to the RIFLE criteria was “Injury.”

Clinical characteristics and laboratory findings of the 3 patients under prevalent hemodialysis who developed sCMP are listed in Tables 3 and 4. Two patients were women and the age range was 54 to 68 years. All 3 patients had diabetes mellitus and hypertension, and 1 patient had atrial fibrillation and bronchial asthma. All 3 patients were under prevalent hemodialysis with anuric state; their weekly Kt/V values were 3.0, 2.58, and 3.52, respectively. At admission, these 3 patients presented with dyspnea. The triggering event for each of these patients was physical stress, which included pneumonia and hypoxemia in 2 patients and infectious colitis in 1 patient. The ECG findings at admission were diffuse T-wave inversion in leads V1 to V6 in 1 patient, ST-segment elevation in the precordial leads in 1 patient, and QTc prolongation (QTc = 504 ms) in 1 patient. Two patients indicated a small increase in cardiac enzyme levels. On echocardiography at presentation, all patients indicated a typical takotsubo pattern with pericardial effusion, and the mean EF was 34.0% (range, 30-37%). Two patients were evaluated with follow-up echocardiography and showed improvement of left ventricular function with a mean EF of 55% within a few weeks (range, 1-2 weeks). The other patient underwent CAG and had no evidence of obstructive epicardial coronary artery disease. Because pericardial effusion was detected in all patients and 2 of them had uremic encephalopathy, these 3 patients were treated with intensive hemodialysis for suspected under-dialysis status. All the patients were treated with conventional hemodialysis and did not exhibit any complications or recurrence of sCMP during the last examination performed in the nephrology department.

**Table 3:** Clinical characteristics of patients under prevalent hemodialysis with stress-induced cardiomyopathy

**Patient No.**	**Underlying disease**	**Weekly Kt/V**	**Presenting symptom**	**Triggering event**	**Renal replacement therapy**	**Outcomes**
5	ESRD, DM, HT	3.0	Dyspnea	Pneumonia	Intensive HD	No recur of sCMP
6	ESRD, DM, HT	2.58	Dyspnea	Infectious colitis	Intensive HD	No recur of sCMP
7	ESRD, DM, HT, AF, Asthma	3.52	Dyspnea	Pneumonia	Intensive HD	No recur of sCMP

*ESRD* end-stage renal disease, *DM* diabetes mellitus, *HT* hypertension, *AF* atrial fibrillation, *HD* hemodialysis, *sCMP* stress-induced cardiomyopathy.

**Table 4:** ECG characteristics, laboratory data, echocardiographic studies, cardiac catheterization data of patients under prevalent hemodialysis with stress-induced cardiomyopathy

**Patient No.**	**ECG abnormality**	**Echocardiographic abnormality at presentation**	**Peak troponin I (ng/mL)**	**Coronary angiography**	**EF at follow-up**	**hs-CRP (mg/dL)**
5	ST-segment elevation	Typical takotsubo, Pericardial effusion, EF = 35%	11.61	NA	51%	7.13
6	T -wave inversion	Typical takotsubo, Pericardial effusion, EF = 37%	4.73	Normal	NA	11.84
7	QTc prolongation	Typical takotsubo, Pericardial effusion, EF = 30%	0.28	NA	58%	17.42

*ECG* electrocardiography, *EF* ejection fraction, *NA* not available, *hs-CRP* high sensitive C-reactive protein.

## Discussion and conclusions

The cases described in this study illustrate that sCMP can occur in patients with renal dysfunction, including end-stage renal disease (ESRD) or AKI. Although sCMP was initially described in Japanese patients, there have been several cases reported in other parts of the world. It is characterized by transient hypokinesia of the apical portion of the left ventricle with compensatory hyperkinesia of the basal walls, resulting in apical ballooning that gives the heart the appearance of a Japanese octopus trap or “takotsubo.” This syndrome mimics an acute coronary syndrome as ECG often reveals ST-segment elevations or T-wave inversions in the precordial leads. The levels of cardiac enzymes are elevated, but angiography shows normal coronary arteries or only minor disease inconsistent with the ECG changes. In previous studies, 39%-80% of patients provide a history of a recent severe emotional or physical stressor [1,2]. Interestingly, the stressor in all of our patients was physical in nature. The exact mechanism and pathophysiology of sCMP is unknown. There have been a number of theories suggested so far, with catecholamine-induced cardiotoxicity being the most widely supported hypothesis [8]. Plasma catecholamine concentrations at the time of onset were increased in patients with sCMP when compared to patients with myocardial infarction [9]. Exogenous administration of catecholamines and pheochromocytoma have been reported to cause typical features of sCMP adding more evidence to this theory [10,11]. During stressful conditions, the sympathetic nervous system is activated, and this may influence myocardial contraction. In addition, a different distribution of sympathetic nerves in the myocardium may also be associated with the peculiar wall-motion abnormality. It has been known that sympathetic nervous system hyperactivity is observed in patients with ESRD. Activation of the sympathetic nervous system, assessed using plasma norepinephrine levels, is observed in patients with ESRD treated with HD and in those with early ESRD without HD treatment; the plasma norepinephrine levels in these patients is significantly higher than those in blood pressure-matched and body mass index-matched hypertensive patients or healthy normotensive subjects [12]. Inflammation and oxidative stress play an important role in the pathogenesis of microvascular dysfunction [13,14]. Recent study found raised C-reactive protein and leucocyte levels in correlation with increased norepinephrine concentration in sCMP patients [15]. It has been demonstrated that vitamin E levels, known to act as a chain-breaking antioxidant that prevents the propagation of free radical reactions, are lower in dialysis patients compared to controls, indicating that there is a significant level of oxidative stress in chronic renal patients [16]. In addition, after dialysis, because of the removal of the antioxidants, an imbalance between oxidants and antioxidants occurs, these instability in the balance of oxidants and antioxidants may be the major cause of cellular oxidative damage. Thus, it can be presumed that, given the conditions of sympathetic nervous hyperactivity and imbalance between oxidants and antioxidants in ESRD patients, triggering of stress might induce sCMP more frequently in these patients than in healthy subjects. Further, all 3 of our patients under maintenance hemodialysis were suspected to have an under-dialysis status because of pericardial effusion and uremic encephalopathy. Therefore, we can presume that the under-dialysis status itself may be a triggering factor for sCMP as a stressor, although further studies are needed to confirm these associations.

Various patterns of sCMP have been recently recognized. In the inverted takotusbo pattern, which is a variant of sCMP, the left ventricular wall exhibits akinesia of the base and hyperdynamic apex [17,18]. This pattern has been known to present at an earlier age compared with other types of sCMP and has been triggered by emotional or physical stress in all patients [18]. This difference in presentation has been attributed to differences in the sympathetic innervations of the heart with the abundance of adrenoreceptors at the apex compared with the base. One of our patients had an inverted takotusbo pattern in initial echocardiography. As mentioned earlier, the most common finding on the admission ECG was ST-segment elevation. In recent study, the classic ECG features included ST elevation (56%), T wave inversion (39%) and pathological Q waves (32%) [19]. Arrhythmia has been reported in 43.5% of cases with ventricular fibrillation, torsade des pointes, ventricular tachycardia and prolonged QTc being the most common and life-threatening arrhythmias occurring in up to 5.7%. New left and right bundle-branch block on the presenting ECG has been reported. The QTc interval on the presenting ECG ranged from a mean of 450 ms to 501 ms [8]. One of our patients had prolonged QTc interval (501 ms), and he died because of recurrent ventricular tachycardia.

Recently, as the clinical spectrum of sCMP became broader and the disease entity was better understood, it was suggested that it is impractical to routinely perform invasive coronary angiography for definitive diagnosis of sCMP. In fact, current criteria for confirming sCMP include invasive coronary angiography, to exclude the possibility of obstructive coronary artery disease or acute plaque rupture. A recent study demonstrated that a combination of typical LV morphologic change of sCMP and noninvasive demonstration of the absence of significant plaque rupture can be possible alternative diagnostic criteria [20]. However, these modified criteria require further evaluation. One of the patients in the present study died during hospitalization; the death was attributed to recurrent ventricular tachycardia. The in-hospital mortality due to sCMP ranges from 0 to 8%. In view of the associated mortality, the overall outcome is better than that of patients with myocardial infarction. Recurrence of this syndrome is rare and infrequent. In the largest series so far, which consisted of 88 patients, a recurrence rate of 2.7% was reported [21]. In our patients, there has been no recurrence after a mean duration of 11 months.

Cardiac diseases are independently associated with deterioration in kidney function and progression of existing kidney diseases [22]. Conversely, both decrease in glomerular filtration rate and proteinuria are independent risk factors for the development of cardiovascular disease [23]. CRS is a disorder of the heart and kidney, whereby acute or long-term dysfunction in 1 of these organs may induce acute or long-term dysfunction of the other. The 2 organs are connected to regulate blood pressure, vascular tone, diuresis, natriuresis, peripheral tissue perfusion and oxygenation. Recently, a new classification of CRS has been developed to reflect the likely primary and secondary pathology and time-frame [24]. Type 1 CRS consists of an abrupt worsening of cardiac function leading to AKI. Proposed pathophysiologic mechanisms include reduced transglomerular pressure, elevated renal interstitial pressure, myogenic and neural reflexes, activation of sympathetic nervous and renin-angiotensin-aldosterone systems, non-osmotic release of arginine vasopressin, local production of endothelin, and enhanced proinflammatory pathways. Type 2 CRS comprises chronic abnormalities in cardiac function causing progressive chronic kidney disease. Accelerated renal cell apoptosis and replacement fibrosis is considered to be the dominant mechanism. Type 3 CRS reflects an abrupt worsening of renal function causing acute cardiac dysfunction. This syndrome is precipitated by salt and water overload, acute uremic myocyte dysfunction, and neurohormonal dysregulation. Type 4 CRS comprises a state of chronic kidney disease that contributes to a decline in cardiac function. Cardiac myocyte dysfunction and fibrosis is believed to be the predominant pathophysiologic mechanism. Type 5 CRS reflects a systemic condition such as sepsis causing both cardiac and renal dysfunction. In this type, the predominant pathophysiological disturbance is microcirculatory dysfunction as a result of acutely abnormal immune cell signaling, catecholamine cellular toxicity, and enzymatic activation which result in simultaneous organ injury often extending beyond both the heart and the kidneys. The use of this classification can help physicians characterize groups of patients and provides the rationale for specific management strategies.

It could be presumed that sCMP and CRS share the comparable mechanism regarding the sympathetic nervous system activation, inflammation and oxidative stress in the pathogenesis of microvascular dysfunction. In our patients, 4 had AKI (injury, failure, and ESRD in 1 each) associated with sCMP. Because the serum creatinine level was already increased at the time that sCMP was diagnosed, we could not appropriately classify the patients according to the CRS classification; instead, we divided them into acute CRS (type 1 or type 3) depending on the clinical course or CRS type 5 regarding the sepsis due to infectious triggering factors.

Two (50%) of the patients with AKI were treated with CRRT to manage the congestive symptoms and correct the abnormalities in electrolyte levels and acid–base status, accompanying hemodynamic instability. In fact, it has been known that the coexistence of heart and kidney failure in the same subject is associated with an extremely bad prognosis [25]. In patients with acute heart failure, an acute increase in serum creatinine level of >0.3 mg/dL is associated with longer hospitalization time, increased mortality, and more frequent re-admissions [26]. Although diuretics have been the mainstay of treatment, they are often unsuccessful in reversing the vicious cycle of volume overload, worsening cardiac function, and azotemia. Renal replacement therapy (RRT) in the form of isolated or continuous ultrafiltration, with or without a component of solute clearance (hemofiltration or hemodialysis), has been increasingly utilized as a therapeutic tool in this setting. A previous study examined the isolated ultrafiltration strategy in 100 patients with hypervolemic congestive heart failure compared with the intravenous diuretics strategy adopted in 100 patients. It demonstrated greater weight loss, decreased need for vasoactive drugs, and fewer rehospitalizations and emergency department visit in the ultrafiltration group, but no difference in mortality [27]. Although the clinical necessity of RRT in cases of life-threatening hyperkalemia or acidemia with AKI is clear, the adjunctive role of RRT (hemofiltration or hemodialysis) in the treatment of acute decompensated heart failure is not obvious. A previous study suggested that because severe hypocalcemia is a potential cause of reversible heart failure and acute or acute on chronic renal dysfunction is often hypocalcemic, correction of hypocalcaemia via continuous venovenous hemofiltration might improve cardiac function in acute decompensated heart failure [28]. However, further research examining the use of RRT in CRS is necessary.

Our current study has some limitations. First, the number of patients is small. Second, significant numbers of patients did not meet the current definition of classic sCMP. As it was not possible to perform invasive coronary angiography in 5 patients, we demonstrated the absence of coronary artery obstruction showing recovery of wall motion abnormality and improvement of left ventricular function to perform follow-up echocardiography. Third, we could not present the exact relevance of sCMP and CRS. Further investigation is necessary to overcome these limitations.

In conclusion, these cases showed that concomitant sCMP can occur in patients with renal dysfunction including ESRD or AKI, especially associated with CRS. Therefore, the renal function of any patient with sCMP should be closely monitored to facilitate the early diagnosis of AKI. ESRD patient who develops sCMP could be considered to be under-dialyzed, but further studies are needed to confirm this.

## Competing interests

The authors declare that they have no competing interests.

## Authors’ contributions

EYS contributed substantially to the design and conception of the manuscript. MJS collected the clinical data, analyzed data, and wrote the manuscript. HR, IYK, and BYY was involved in the interpretation of the data. SHS, DWL, and SBL contributed substantially to the writing of the manuscript. ISK revised the manuscript. JHC collected the echocardiographic data. All authors read and approved the final manuscript.

## Pre-publication history

The pre-publication history for this paper can be accessed here:

http://www.biomedcentral.com/1471-2369/14/213/prepub

## References

[B1] Previtali M Repetto A Panigada S Camporotondo R Tavazzi L Left ventricular apical ballooning syndrome: prevalence, clinical characteristics and pathogenetic mechanisms in a European population Int J Cardiol 2009 134 91 96 10.1016/j.ijcard.2008.01.037 18508143

[B2] Sharkey SW Windenburg DC Lesser JR Maron MS Hauser RG Lesser JN Natural history and expansive clinical profile of stress (Tako-Tsubo) Cardiomyopathy J Am Coll Cardiol 2010 55 333 341 10.1016/j.jacc.2009.08.057 20117439

[B3] Copetti R Gonano C Colombo T Cattarossi L Inverted Takotsubo pattern Resuscitation 2007 74 394 17521794 10.1016/j.resuscitation.2007.04.009

[B4] Movahed MR Mostafizi K Reverse or inverted left ventricular apical ballooning syndrome (reverse Takotsubo cardiomyopathy) in a young woman in the setting of amphetamine use Echocardiography 2008 25 429 432 10.1111/j.1540-8175.2007.00604.x 18177388

[B5] Bongartz LG Cramer MJ Doevendans PA Joles JA Braam B The severe cardiorenal syndrome: ‘Guyton revisited’ Eur Heart J 2005 26 11 17 15615794 10.1093/eurheartj/ehi020

[B6] Arroyo D Panizo N Verdalles U Vázquez-Álvarez ME Barraca D Quiroga B Acute kidney failure in the context of a Tako-Tsubo syndrome Nefrologia 2011 31 493 494 21738255 10.3265/Nefrologia.pre2011.Apr.10879

[B7] Ricci Z Cruz D Ronco C The RIFLE criteria and mortality in acute kidney injury: a systematic review Kidney Int 2008 73 538 546 10.1038/sj.ki.5002743 18160961

[B8] Bybee KA Kara T Prasad A Lerman A Barsness GW Wright RS Systematic review: transient left ventricular apical ballooning: a syndrome that mimics ST segment elevation myocardial infarction Ann Intern Med 2004 141 858 865 10.7326/0003-4819-141-11-200412070-00010 15583228

[B9] Wittstein IS Thiemann DR Lima JA Baughman KL Schulman SP Gerstenblith G Neurohumoral features of myocardial stunning due to sudden emotional stress N Engl J Med 2005 352 539 548 10.1056/NEJMoa043046 15703419

[B10] Abraham J Mudd JO Kapur NK Klein K Champion HC Wittstein IS Stress cardiomyopathy after intravenous administration of catecholamines and beta-receptor agonists J Am Coll Cardiol 2009 53 1320 1325 10.1016/j.jacc.2009.02.020 19358948

[B11] Marcovitz PA Czako P Rosenblatt S Billecke SS Pheochromocytoma presenting with takotsubo syndrome J Interv Cardiol 2010 23 437 442 10.1111/j.1540-8183.2010.00551.x 21029177

[B12] Masuo K Mikami H Ogihara T Tuck M Hormonal mechanisms in blood pressure reduction during hemodialysis in patients with chronic renal failure Hypertens Res 1995 18 Suppl 1 S201 S203 8529063 10.1291/hypres.18.supplementi_s201

[B13] Ueyama T Kawabe T Hano T Tsuruo Y Ueda K Ichinose M Upregulation of heme oxygenase-1 in an animal model of Takotsubo cardiomyopathy Circ J 2009 73 1141 1146 10.1253/circj.CJ-08-0988 19372624

[B14] Nef HM Möllmann H Kostin S Troidl C Voss S Weber M Intraindividual structural analysis in the acute phase and after functional recovery Eur Heart J 2007 28 2456 2464 10.1093/eurheartj/ehl570 17395683

[B15] Morel O Sauer F Imperiale A Cimarelli S Blondet C Jesel L Importance of inflammation and neurohumoral activation in Takotsubo cardiomyopathy J Card Fail 2009 15 206 213 10.1016/j.cardfail.2008.10.031 19327622

[B16] Kadkhodaee M Hemmati M Zahmatkesh M Ghaznavi R Mirershadi F Mahdavi-Mazde M Assessment of plasma antioxidant status in hemodialysis patients Ther Apher Dial 2008 12 147 151 10.1111/j.1744-9987.2008.00561.x 18387164

[B17] Lee YP Poh KK Lee CH Tan HC Razak A Chia BL Diverse clinical spectrum of stress-induced cardiomyopathy Int J Cardiol 2009 133 272 275 10.1016/j.ijcard.2007.11.039 18190984

[B18] Ramaraj R Movahed MR Reverse or inverted takotsubo cardiomyopathy (reverse left ventricular apical ballooning syndrome) presents at a younger age compared with the mid or apical variant and is always associated with triggering stress Congest Heart Fail 2010 16 284 286 10.1111/j.1751-7133.2010.00188.x 21091614

[B19] Milinis K Fisher M Takotsubo cardiomyopathy: pathophysiology and treatment Postgrad Med J 2012 88 530 538 10.1136/postgradmedj-2012-130761 22647668

[B20] Lee PH Song JK Sun BJ Choi HO Seo JS Na JO Outcomes of patients with stress-induced cardiomyopathy diagnosed by echocardiography in a tertiary referral hospital J Am Soc Echocardiogr 2010 23 766 771 10.1016/j.echo.2010.05.002 20620862

[B21] Elian D Osherov A Matetzky S Hod H Guetta V Feinberg MS Left ventricular apical ballooning: not an uncommon variant of acute myocardial infarction in women Clinical Cardiology 2006 29 9 12 10.1002/clc.4960290104 16477771 PMC6654087

[B22] Elsayed EF Tighiouart H Griffith J Kurth T Levey AS Salem D Cardiovascular disease and subsequent kidney disease Arch Intern Med 2007 167 1130 1136 10.1001/archinte.167.11.1130 17563020

[B23] Sarnak MJ Levey AS Schoolwerth AC Coresh J Culleton B Hamm LL Kidney disease as a risk factor for development of cardiovascular disease: a statement from the American Heart Association Councils on Kidney in Cardiovascular Disease, High Blood Pressure Research, Clinical Cardiology, and Epidemiology and Prevention Circulation 2003 108 2154 2169 10.1161/01.CIR.0000095676.90936.80 14581387

[B24] Ronco C House AA Haapio M Cardiorenal syndrome: refining the definition of a complex symbiosis gone wrong Intensive Care Med 2008 34 957 962 10.1007/s00134-008-1017-8 18251008

[B25] van Kimmenade RR Januzzi JL Jr Baggish AL Lainchbury JG Bayes-Genis A Richards AM Amino-terminal pro-brain natriuretic Peptide, renal function, and outcomes in acute heart failure: redefining the cardiorenal interaction? J Am Coll Cardiol 2006 48 1621 1627 17045898 10.1016/j.jacc.2006.06.056

[B26] Damman K Jaarsma T Voors AA Navis G Hillege HL van Veldhuisen DJ Both in- and out-hospital worsening of renal function predict outcome in patients with heart failure: results from the Coordinating Study Evaluating Outcome of Advising and Counseling in Heart Failure (COACH) Eur J Heart Fail 2009 11 847 854 10.1093/eurjhf/hfp108 19696057

[B27] Costanzo MR Guglin ME Saltzberg MT Jessup ML Bart BA Teerlink JR Ultrafiltration versus intravenous diuretics for patients hospitalized for acute decompensated heart failure J Am Coll Cardiol 2007 49 675 683 10.1016/j.jacc.2006.07.073 17291932

[B28] Udani SM Murray PT The use of renal replacement therapy in acute decompensated heart failure Semin Dial 2009 22 173 179 10.1111/j.1525-139X.2008.00542.x 19426424

